# Utilization of sewage sludge in the manufacture of lightweight aggregate

**DOI:** 10.1007/s10661-015-5010-8

**Published:** 2015-12-03

**Authors:** Małgorzata Franus, Danuta Barnat-Hunek, Magdalena Wdowin

**Affiliations:** Civil Engineering and Architecture Faculty, Lublin University of Technology, Nadbystrzycka 40, 20-618 Lublin, Poland; The Mineral and Energy Economy Research Institute of the Polish Academy of Sciences, Wybickiego 7, 31-261 Kraków, Poland

**Keywords:** Sewage sludge, Lightweight aggregates, Heavy metals, Leachability test, Physical and mechanical properties of lightweight aggregates

## Abstract

This paper presents a comprehensive study on the possibility of sewage sludge management in a sintered ceramic material such as a lightweight aggregate. Made from clay and sludge lightweight aggregates were sintered at two temperatures: 1100 °C (name of sample LWA1) and 1150 °C (name of sample LWA2). Physical and mechanical properties indicate that the resulting expanded clay aggregate containing sludge meets the basic requirements for lightweight aggregates. The presence of sludge supports the swelling of the raw material, thereby causing an increase in the porosity of aggregates. The LWA2 has a lower value of bulk particle density (0.414 g/cm^3^), apparent particle density (0.87 g/cm^3^), and dry particle density (2.59 g/cm^3^) than it is in the case of LWA1 where these parameters were as follows: bulk particle density 0.685 g/cm^3^, apparent particle density 1.05 g/cm^3^, and dry particle density 2.69 g/cm^3^. Water absorption and porosity of LWA1 (*WA* = 14.4 %, *P* = 60 %) are lower than the LWA2 (*WA* = 16.2 % and *P* = 66 %). This is due to the higher heating temperature of granules which make the waste gases, liberating them from the decomposition of organic sewage sludge. The compressive strength of LWA2 aggregate is 4.64 MPa and for LWA1 is 0.79 MPa. Results of leaching tests of heavy metals from examined aggregates have shown that insoluble metal compounds are placed in silicate and aluminosilicate structure of the starting materials (clays and sludges), whereas soluble substances formed crystalline skeleton of the aggregates. The thermal synthesis of lightweight aggregates from clay and sludge mixture is a waste-free method of their development.

## Introduction

Sewage sludge is a by-product of the process of municipal wastewater treatment (Tuan et al. 2013). An increase in the requirements for quality of wastewater discharged to the environment results in the production of a higher quantity of sewage sludge generated in the process of wastewater treatment. The method of sewage sludge management in Poland is subject to the regulation on waste management (Polish Act on Waste of 27 April 2001; Polish Ordinance of the Minister of Environment of 13 July 2011; Polish Regulation of the Minister of Environment of 24 July 2006) as well as to other acts and regulations resulting from the transposition of European Union legislation to the national law. These regulations define the minimum standards regarding the conditions which must be met when applying sewage sludge in agriculture. In particular, they determine the limits for heavy metals in sewage sludge and soils where these deposits can be used. Municipal sewage sludge is characterized by specific soil and fertilizer values. The content of nutrients and trace elements in sewage sludge determine their use in nature (Jordán et al. 2005; Montero et al. 2009; Teo et al. 2014). The organic substances in sewage sludge improve the physical properties of the soil, thus increasing its sorption capacity. In contrast, nutrients (N, P, K) provide for the proper development of plants and soil microorganisms’ mineral substances. Such a method of utilization of sewage sludge is often limited or prevented in the presence of the pathogenic microorganisms and heavy metals, which in trace amounts are essential for plant and animal life. However, heavy metals in higher quantities are toxic and carcinogenic and can also accumulate in living organisms (Ahmaruzzaman 2011; Franus and Bandura 2014). The presence of heavy metals in sewage sludge, especially in large quantities, results from the participation of industrial wastewater (e.g., tanneries, paint, steel) in the overall mass of municipal sewage (Bingham and Hand 2006; Chang et al. 2007).

Currently, the disposal of waste is handled in the following ways: land or sea dumping, recycling, incineration, or utilization for agriculture purposes (Rubli et al. 2000; Bagreev and Bandosz 2004; Werle and Wilk 2010). However, the incineration of sewage sludge causes the emission of harmful compounds resulting in public opposition and requires high investment for the flue gas treatment section. In addition, some by-products in the form of ashes are generated as a result of the incineration process (Fytili and Zabaniotou 2008). Therefore, the recycling of the wastes as raw materials for various valuable products is the most desirable and environment-friendly solution to properly manage the huge amount of the waste (Kante et al. 2008; Teo et al. 2014). One way to manage sewage sludge is their use (similar as in the case of fly ash (Wainwright and Cresswell 2001; Ramamurthy and Harikrishnan 2006; Sarabèr et al. 2012; Franus et al. 2014; Wdowin et al. 2014). in the production of building materials (Weng et al. 2003; Liew et al. 2004; Jordán et al. 2005; Montero et al. 2009) but in particular lightweight aggregates (LWAs) (Bingham and Hand 2006; Zou et al. 2009; Gonzáles-Corrochano et al. 2010; Qi et al. 2010; Liu et al. 2012). Application of sewage sludge to production of lightweight aggregates or ceramics is a promising option as this solution avoids the secondary pollution as well as increases the value of sludge by converting it into useful material (Xu et al. 2010; Quina et al. 2014).

LWAs are granular and porous materials applied in architecture, gardening, and geotechnics (Bodycomb and Stokowski 2000; Cheeseman et al. 2005; Fakhfakh et al. 2007).

They can be used for wall concrete blocks to reduce the weight of a building with high acoustics and fire resistance. LWAs are a component of building materials such as prefabricated structural units and structural lightweight concretes — especially in high-rise buildings, as well as track ballasts and road coatings (Ducman and Mitrič 2009).

The purpose of this study is to utilize sewage sludge coming from municipal-industrial wastewater treatment plants containing significant quantities of heavy metals, to obtain an environmentally friendly lightweight aggregates.

## Materials and methods

### Characterization of starting materials

Sewage sludge from the municipal sewage treatment plant “Hajdów” in Lublin, Poland, and clay from the “Budy Mszczonowskie” deposit in Poland were testing materials for the production of LWA. The Hajdów sewage treatment plant is a mechanical-biological sewage treatment plant dealing with municipal sewage as well as a part of industrial waste. Sludge was taken from the mechanical dewatering station in which the post-fermentation sludge hydration was decreased, and hence, its volume was reduced. The analysis of physical-chemical properties of sewage sludge was carried out according to standards EN-ISO 12176:^2004^, EN-ISO 6060:^2006^, and EN-ISO 9963-1:^1995^. Ortho-phosphate properties were examined in accordance with ISO/FDIS 15681-1, and the TOC was determined using the Shimadzu TOC-5050 total organic carbon analyzer. The chemical composition of sewage sludge was determined using the atomic emission spectroscopy (AES), the Jarrell ASH Enviro model, inductively coupled plasma mass spectrometry (ICP/MS), and the Parkin Elmer Elan 6000 model. The chemical composition of clay was determined by the XRF method. The Philips spectrometer PW 1404 was used, and the induction source was constituted by a XRD lamp with a double anode (Cr–Au) with a maximum power of about 3 kW.

### Thermogravimetric and differential thermal analysis

The thermal analysis (differential thermal analysis (DTA)/thermogravimetric (TG)) for the clay and sewage sludge was performed using STA 449 F3 Jupiter Netzsch apparatus coupled with a quadrupole mass QMS 403 C Aeolos spectrometer and FTIR Bruker spectrometer.

### Mineralogy and microstructural analysis

The mineral composition of the samples (substrates and products) was determined by powder X-ray diffraction method, using a Panalytical X’pert APD diffractometer (with a PW 3020 goniometer), a Cu lamp, and a graphite monochromator. The analysis was performed at the angle range of 5–65 2θ. Philips X’Pert High Score software was used to process the diffraction data. The identification of mineral phases was based on the PDF-2 release 2010 database formalized by the ICDD. The morphological forms were determined using scanning electron microscope (SEM) FEI Quanta 250 FEG.

### Preparation of lightweight aggregates (LWA1, LWA2)

Both clay and sewage sludge were dried at a temperature of 105 °C to a state of oven-dry and then grounded to a grain size of <0.5 mm.

To obtain a lightweight aggregate as the basic material, a clay from the Budy Mszczonowskie deposit was used as well as sewage sludge. Both starting materials were dried at 105 °C and then milled in a ball mill to a fraction with a diameter of <0.5 mm. Then, the sewage sludge in the amount of 10 wt.% (i.e., 10 g) was added to the clay in the amount of 90 wt.% (i.e., 90 g) and mixed. In order to obtain a plastic, mass water (40 mL) was added to the dry reactants which was suitable for forming by the hands the granules with a diameter of about 16 mm.

The processes of mixing and extrusion of clay-sludge mixtures are essential to obtain an appropriate plastic green matter in order to avoid the formation of segregate agglomerates without possibility to encapsulate them within the vitreous matrix of the ceramic product (Cusidó and Cremades 2012).

**Fig. 1:**
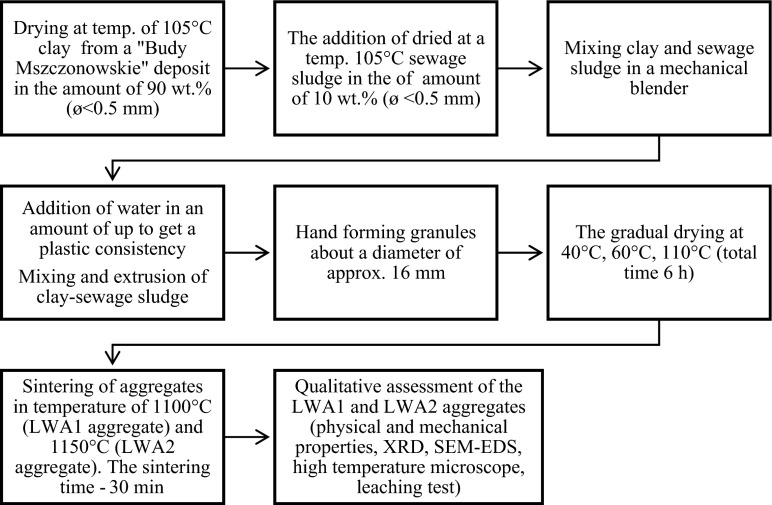
The diagram showing the procedure for disposing of sewage sludge to obtain lightweight aggregates of LWA1 and LWA2

The derived granules were dried in stages: first at 40 °C, second at 60 °C, and third at 110 °C. The drying time of the granules at a given temperature was 2 h. After drying, tested material was sintered in a chamber furnace at a temperature of 1100 and 1150 °C for half an hour (Fig. 1). The increase in temperature was 5 °C/min. Samples after sintering were left in the oven for their cool down to a temperature of 100 °C. The obtained aggregates of clay and sludge in the article were marked as LWA1—the aggregate of clay and sludge sintered at 1100 °C—and LWA2—the aggregate of clay and sludge sintered at 1150 °C.

### Physical and mechanical properties of lightweight aggregates (LWA1, LWA2)

The particle density and water absorption were determined using an established procedure described by EN-ISO 1097-6:2000b, while bulk density and void percentage were determined according to EN-ISO 1097-3:2000a standards. The parameters were calculated according to Eqs. (1, 2, and 3), respectively:Apparent particle density:

1 $$ {P}_a=\frac{M_4}{M_4-\left({M}_2-{M}_3\right)} $$

Dry particle density:

2 $$ {P}_d=\frac{M_4}{M_1-\left({M}_2-{M}_3\right)} $$

Water absorption:

3 $$ W{A}_{24h}=\frac{100\left({M}_1-{M}_4\right)}{M_4} $$

where

*M*_*1*_: mass (g) of saturated pellets on the dry surface
*M*_*2*_: mass (g) in the pycnometer containing saturated pellets and water
*M*_*3*_: mass (g) in the pycnometer containing water
*M*_*4*_: mass (g) of the dry sample, prior to immersion in water.

The bulk density of lightweight aggregates was calculated according to the following formula:

4 $$ {P}_b=\frac{m_2-{m}_1}{v} $$

where

*ρ*_*b*_: bulk density (g/cm^3^)
*m*_*1*_: weight of the empty container (g)
*m*_*2*_: weight of the container and the test sample (g)
*V*: volume of the container (cm^3^).

The void percentage (*H*) (air space between aggregates in a container) and porosity (*P*) were calculated according to the formula

5 $$ H=\frac{100\cdotp \left({p}_d-{p}_b\right)}{P_d} $$

6 $$ P=\frac{P_d-{P}_a}{p_d} $$

where

*H*: void percentage (%)
*P*: porosity (%)
*ρ*_*a*_, *ρ*_*b*_: apparent and bulk density
*ρ*_*d*_: dry particle density of the sample.

Compressive strength was measured according to the UNE-EN 13055-1 standard. The force needed to recess the piston to a predetermined depth in a cylinder filled with compacted aggregate is a measure of the resistance. Compressive strength was calculated according to the equation:

7 $$ {C}_a=L+\frac{F}{A} $$

where

*C*_*a*_: compressive strength (N/mm^2^ or MPa)
*L*: force exerted by the piston (N)
*F*: force needed to the piston cavity (N)
*A*: piston area (mm^2^).

Frost resistance of lightweight aggregate was determined according to the EN-ISO 1367-1 standard. It is the maximum allowable percentage of loss in mass of the aggregate soaked with water and subjected to cyclic freezing to −17.5 °C (10 cycles) and thawing to 20 °C.

### High-temperature microscope

By using a high-temperature microscope, the temperatures of the commercial aggregate (LWA0), LWA1, and LWA2 were determined. The crushed samples’ lightweight aggregates combined with water and pressed in a hand press (to obtain a cylindrical shape, 3 mm in diameter and 3 mm high) were subjected to thermal processing. Temperature gain in the high-temperature microscope was set up to 1100–30 °C/1 min and over 1100–10 °C/1 min. The experiment was performed in an oxidation atmosphere.

### Leaching test

Due to the presence of heavy metals in sewage sludge, their leachability from samples LWA1 and LWA2 was determined. Leachates are the eluates formed by reaction, draining, or filtration of materials contained in the waste (Cusidó and Cremades 2012). These are substances occurring in the form of a solution or suspension, which can penetrate groundwater or soil, posing a risk to the environment. In this case, leachates from the sintering materials (lightweight aggregate) made with sewage sludges are primary heavy metals that were present in the waste.

Due to the lack of standardization of leachability of heavy metals, there are many methods for determination (Van der Sloot 2003; Gonzáles-Corrochano et al. 2009a, b). One of the methods used for leaching hazardous substances such as cadmium, chromium, copper, nickel, lead, and zinc was the method based on the ISO-ISO 1744-3 standard. Another method to determine the leachability of heavy metals was the maximum leaching (ML) method which is applied to obtain the immobilization level of heavy metals from building materials (Rankers and Hohberg 1991; Stegeman and Cot’e 1996; Franus et al. 2011). The ML method determines the maximum possibility of leaching toxins from the matrix in extreme conditions. Distilled water in the quantity of 500 mL, acidified with 1 N HNO_3_ to pH = 4, was added to 5 g of ground-up samples which had a fraction below 125 μm. Samples prepared in this way have been shaken for 5 h, and the pH of solutions throughout the process was kept constant by using 1 N HNO_3_. After leaching, the suspensions were centrifuged. The obtained clear solutions were decanted, and the concentration of heavy metals of the remaining elements was detected with ICP-AES method.

## Results

### Characterization of starting materials

Test results of sewage sludge from the sewage treatment plant Hajdów in Lublin are presented in Table 1. The increased content of heavy metals indicates that the sediments are not suitable for use in agriculture. The high content of organic matter could affect the growth of aggregate porosity.

**Table 1:** Physical and chemical characteristics of sewage sludge from the “Hajdów” wastewater treatment plant

Physical parameters	Average values	Chemical parameters	Average values
Moisture content (%)	80.43	Alkalinity (mg CaCO_3_/L)	750
Dry mass (%)	19.57	Chemical oxygen demand (mg/L)	136,423
Residue on ignition (%)	39.35	Volatile fatty acids (mg/L)	92
Loss on ignition (%)	60.65	Zn(II) (ppm)	230.92
Total organic matter (%)	68	Cu(II) (ppm)	86.6
Density (g/mL)	0.795	Cr(II) (ppm)	14.51
pH	7.68	Ni(II) (ppm)	8.19
		Pb(II) (ppm)	4.94
		P-PO_4_^3−^ (mg/L)	129

The analysis of chemical composition of clay shows a high content of SiO_2_ (68.5 %), Al_2_O_3_ (15.3 %), Fe_2_O_3_ (6.6 %), K_2_O (2.37 %), Na_2_O (1.91 %), CaO (0.32 %), TiO_2_ (0.22 %), BaO (0.018 %), MnO (0.012 %), and ZnO (0.03 %). The presence of these main compounds in raw materials of the ceramic sinter contributes to the formation of the liquid phase affecting the viscosity of the clay and decreasing the temperature of swelling (Riley 1950).

### Thermogravimetric and differential thermal analysis (DTA/TG)

**Fig. 2:**
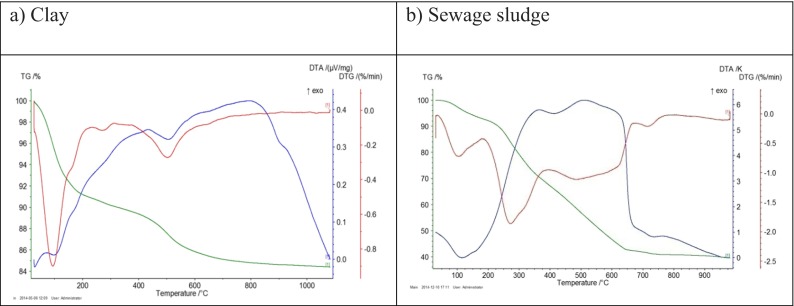
DTA (TG) curves for the clay from Mszczonów (**a**) and sewage sludge (**b**)

On the DTA curve of the analyzed clay (Fig. 2a), there are three endothermic reactions observed which are within the limits of 100, 250, and 500 °C. The first endothermic reaction is associated with the loss of water adsorbed by the surface (moisture). The second indicates the presence of small quantities of iron oxides and hydroxides in the analyzed material. The last is connected with the low-temperature conversion of quartz into a high-temperature quartz. The weight loss of approximately from 15 to 25 % due to dehydroxylation is shown on the TG curve.

There are three areas of the DTA curve in the examined sewage sludge (Fig. 2b). The first involves a clear low-temperature endothermic effect — beginning at 20 °C and ending at 180 °C — and the maximum occurs at 115 °C. An evident weight loss of about 7 % is visible during this effect. The endothermic effect goes into very extensive and intense exothermic effect with two maxima at 360 and 510 °C. Exotherm ends rapidly around 650–670 °C. A significant weight loss of about 50 % is associated with this effect. In the range of 180 to 395 °C, the sample has lost 24.9 wt.%, and at the second step (670 °C), the weight loss of 25.6 wt.% is observed. The thermogram graph suggests that at least two types of organic matter with varying degrees of transformation are present in the sample. Two exothermic peaks and two mass losses confirm this statement. More transformed organic matter begins to decompose (oxidize) at temperatures of 400–450 °C. The end of this process appears in the temperature of 650–670 °C and is characterized by sudden disappearance of exotherm. At the final stage of the heating process (>700 °C), a clear endothermic effect with a maximum at 720 °C, which is associated with a high weight loss, is visible. This is probably related to the thermal dissociation of calcite — and a clear asymmetry and a relatively high reaction temperature suggest such an effect. The loss in mass of 1.52 %, which occurs with dissociation, indicates the CaCO_3_ content of about 3.5 wt.%. After completion of the decomposition of carbonates in the sample, no significant changes occur, although it still loses less than 1 % of its weight.

### Mineralogy and microstructural analysis

**Fig. 3:**
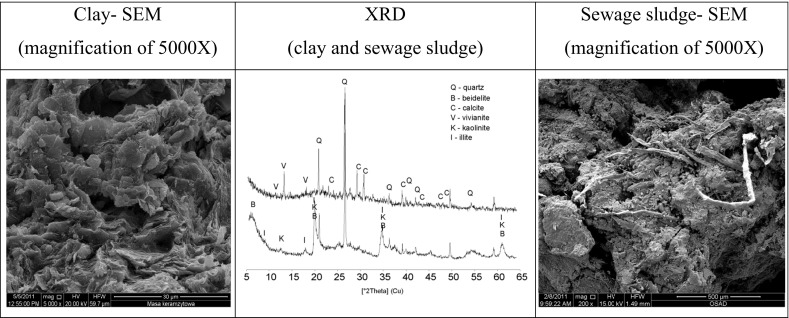
X-ray diffraction (XRD) patterns of clay and sewage sludge and microphotograph (SEM)

The X-ray diffraction analysis was performed to establish the inorganic crystalline composition of sewage sludge and clay (Fig. 3).

The main mineral components of sewage sludge which were identified include calcite (*d*_hkl_ = 3.86, 3.04, 2.28, 1.91 Å), vivianite (*d*_hkl_ = 7.94, 6.70, 4.90 Å), and quartz (*d*_hkl_ = 4.27, 3.34, 2.45, 1.82 Å). The main mineral components of clay are minerals represented by beidelite (*d*_hkl_ = 15.15, 4.44, 2.59, 1.49 Å), illite (*d*_hkl_ = 10.01, 5.02, 4.48, 3.34, 2.59, 1.49 Å), and kaolinite (*d*_hkl_ = 7.14, 4.48, 4.36 Å), which are accompanied by quartz in minor quantities.

### Physical properties of lightweight aggregates (LWA1, LWA2)

Certain physical and mechanical parameters of modified sewage sludge aggregates (LWA1, LWA2) indicate that they meet the requirements specified by the standards (Table 2).

**Table 2:** Physical and mechanical parameters of LWA1 and LWA2 and comparison of them to commercial lightweight aggregates available in Poland

Aggregates	*ρ*_*d*_ (g/cm^3^)	*ρ*_*a*_ (g/cm^3^)	*ρ*_*b*_ (g/cm^3^)	*WA*_*24*_ (%)	*H* (%)	*P* (%)	*F* (%)	*C*_*a*_ (MPa)
LWA1 (1100 °C)	2.69	1.05	0.685	14.4	74	60	0.08	4.64
LWA2 (1150 °C)	2.59	0.87	0.414	16.2	84	66	1	0.79
LIAPOR (import)	–	0.55–1.0	0.25–0.55	10–20	–	–	<1	1.0–4.0
LECA WEBER	–	0.5–0.9	0.25–0.65	30–40	–	–	<1	0.7–4
LWA0 (Mszczonów)	–	0.95–1.1	0.4–0.9	20–30	–	–	<1	2.0–6.0

*ρ*_*b*_ bulk density (g/cm^3^), *ρ*_*a*_ apparent particle density (g/cm^3^), *ρ*_*d*_ dry particle density (g/cm^3^), *WA*_*24*_ water absorption (%), *H* void percent (%), *P* porosity (%), *F* frost resistance (%), *C*_*a*_ compressive strength (MPa)

Bulk density is less than 1.2 g/cm^3^ which indicates that LWA1 and LWA2 are lightweight aggregates. Dry particle density values obtained for all aggregates are less than 2 g/cm^3^; aggregates manufactured in this study are classified as lightweight aggregates according to UNE-EN-13055-1 standard. The generally acceptable water absorption capacity based on a 24-h soaking cycle in water is less than 20 %. Water absorption of LWA1 aggregate is 14.4 %, while for LWA2 aggregate, it is increased to the value of 16.2 %. Slightly lower absorption values of aggregates are due to the presence of a glassy film, which is clearly visible on LWA2 aggregates. This slight increase in water absorption is probably due to the growth in open porosity of grains and bigger heterogeneity of the grain shape. A particle with isolated pores or a vitrified surface tends to absorb little water, whereas the one with connected or open pores will absorb water like a sponge (Huang and Hwang 2007).

Lightweight aggregate LWA2 is of higher porosity (66 %) than the LWA1 (60 %). This is due to the higher heating temperature of granules which make the waste gases, liberating them from the decomposition of organic sewage sludge and increasing the porosity of aggregates. Potential sources of gas evolution in artificial aggregates could be one or more of the following: the clay mineral itself (absorbed as well as chemically held water), calcium carbonate, ferric oxide, and entrapped air (Gonzáles-Corrochano et al. 2009a, b). Frost resistance of aggregate does not exceed 1 %. The aggregate grain did not show any occurrence of cracks after the test.

The compressive strength of LWA2 aggregate is 4.64 MPa and for LWA1 is 0.79 MPa. Significant differences in strength are due to the predominance of the glassy film on the surface of LWA2 aggregate as well as bigger porosity. The presence of pores much larger in size reduces the crushing strength of LWA2 aggregates. LWA1 with less pores have a higher compressive strength than LWA2 with the lowest density (more pores) (Cusidó et al. 2003).

**Fig. 4:**
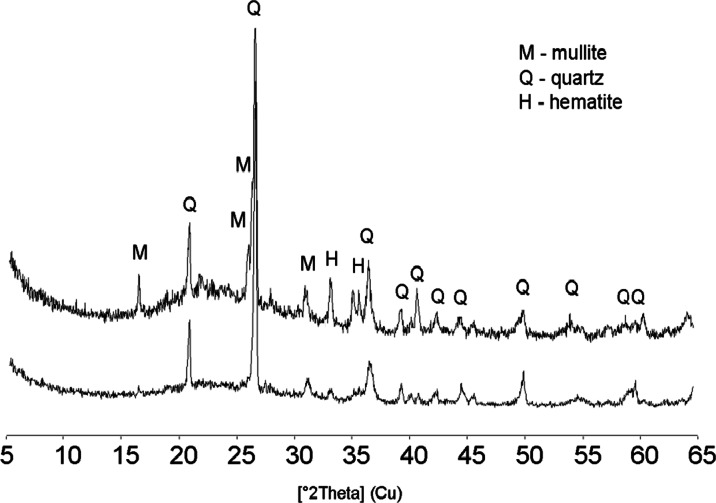
X-ray diffraction (XRD) patterns of LWA1 and LWA2

In temperatures of 1100 and 1150 °C, the organic matter is totally destroyed and is responsible for the high porosity of the microstructure (Huang et al. 2007). The mineral composition of LWA2 lightweight aggregate is dominated by the glassy phase which is recognized by the characteristic diffraction pattern within angle ranges 15–30 (2θ), mullite (*d*_hkl_ = 5.41, 3.43, 3.39, 2.88 Å), hematite (*d*_hkl_ = 2.70, 2.51 Å), and quartz (Fig. 4).

**Fig. 5:**
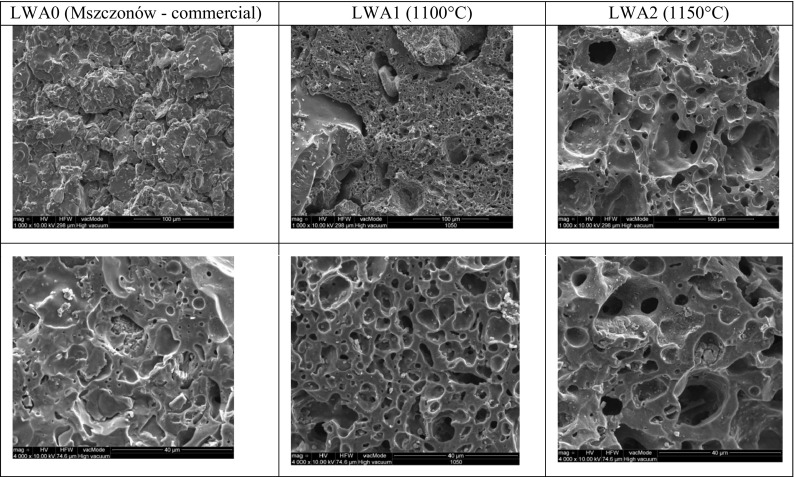
Microstructures of LWA0, LWA1, and LWA2 aggregates at magnification of 4000× and 8000×

The microphotographs from the SEM show the porous structure of commercial aggregate (LWA0), as well as LWA1 and LWA2 (Fig. 5).

The presence of pores in fired aggregates is due to thermal swelling of clay at high temperature where the mineral material reaches the pyroplastic condition, and gases liberating from the raw material have sufficient vapor pressure to increase the volume of the closed pores. Along with the attainment of the pyroplastic condition, there is a solid, liquid, and a gas phase in the sintered material (Weinecke and Faulkner 2002). LWA0 (commercial aggregate) is characteristic of a compact texture, predominantly with pores of 5–18 μm, which are oval and sometimes elongated. Aggregates LWA1 and LWA2 have pores of much larger sizes. This is due to the fact that during the sintering process, components of the organic substance coming from sewage sludge additionally produce gases, which contribute to the formation of pore beads and the creation of a porous structure of aggregate (Wang et al. 2009). This porosity, although affecting the mechanical resistance, gives the lightweight aggregate interesting properties such as low weight and thermal and acoustic insulation. At a higher temperature of firing, a considerable increase in the size of pores is noticed. LWA2 is of a porous structure and has visible pores of a spherical shape which prevail in the fired material. The pore size varies and ranges from 5 μm to over 90 μm. LWA1 aggregate has slightly smaller pores, the size of which ranges in value from 5 to 30 μm. The placement of the pores is quite irregular.

### High-temperature microscope

Observations made using a high-temperature microscope aided the calculation of temperatures of the aggregates. Sewage sludge added to LWA1 and LWA2 aggregates caused an increase in the temperature values of softening, melting, and flowing compared to the LWA0 aggregate (Table 3). The temperature of aggregates sintering showed the threshold of their safe firing, and a wide interval between *T*_3_ and *T*_2_ prevented their deformation.

**Table 3:** Results of fusibility tests

Aggregates	*T*_1_	*T*_2_	*T*_3_	*T*_4_	*T*_5_	*T*_2_-*T*_1_	*T*_3_-*T*_2_
LWA0 (Mszczonów)	718	1100	1187	1405	1441	382	87
LWA1	804	1075	1263	1430	1470	271	188
LWA2	804	1141	1274	1413	1466	337	133

Characteristic temperatures (in °C) of beginning of sintering (*T*_1_), peak sintering (*T*_2_), softening (*T*_3_), melting (*T*_4_), flowing point (*T*_5_), and firing intervals (*T*_2_-*T*_1_ and *T*_3_-*T*_2_)

The temperature of the initial sintering of LWA0 aggregate in relation to LWA1 aggregate is higher. The highest value was obtained for LWA2 and amounts to 1141 °C. The flowing temperature of LWA0 is much higher than the flowing temperatures of aggregates made from sewage sludge, while for LWA2 aggregate, a favorable reduction of a melting temperature was observed (hemispherical point) as compared to the LWA1. The values of temperatures of softening, initial sintering, and flowing of LWA2 are higher than temperature values for LWA1 and LWA0 aggregates. The test samples have a wide temperature interval for LWA0 (718–1100 °C), LWA1 (804–1075 °C), and LWA2 (804–1141 °C). Thus, in determining the thermal characteristics of a given raw material in relation to the standards PN-82/G-04535a, PN-82/G 04535b, BN-81/7001-01a, and BN-81/7001-01b, it is shown that the interval of the firing temperature for each of the tested samples is much higher than 50 °C—something very important for the industrial use of the tested materials.

### Leachability test

Leachability measurements of elements in water extracts showed that the presence of metals is much smaller than is acceptable according to Decree of the Minister of Environment dated 28 January 2009, on the conditions required for the discharge of sewage in water or soil and on substances particularly harmful to the aquatic environment (Table 4). Elements from the aggregate leaching tests indicate their very low mobility. Element concentrations in water extracts have been determined in accordance with EN-ISO 1744:^2002^ and are slightly higher than those specified based on the ML method.

**Table 4:** Leaching test conducted in lightweight aggregates

Method of analysis	Zn(II)		Cu(II)		Cr(III)		Ni(II)		Pb(II)		Ti(IV)		Al(III)		Fe(III)	
Type of aggregate	LWA1	LWA2	LWA1	LWA2	LWA1	LWA2	LWA1	LWA2	LWA1	LWA2	LWA1	LWA2	LWA1	LWA2	LWA1	LWA2
EN-ISO 1744-3	0.015	0.012	<d.l	<d.l	<d.l	<d.l	0.085	0.083	0.067	0.055	0.045	0.038	0.57	0.45	0.76	0.65
Method ML	1.35	0.6	0.94	0.76	0.25	0.08	0.08	0.07	0.07	0.055	0.087	0.074	2.13	0.95	3.67	2.58
Value admissible^a^	<2.0		<2.0		<0.5		<0.1		<0.1		<0.1		<3.0		<10.0	

Leachate concentrations are expressed as mg/L

*<d.l* below detected limit of ICP-AES analysis

^a^Requirements according to the Regulation of the Minister of Environment dated 28 January 2009 on the required conditions at the disposal of sewage in water or soil and on substances particularly harmful to the aquatic environment

The sintering of aggregates at a higher temperature (1100 and 1150 °C) reduces the leaching of the analyzed elements, particularly Zn, Cu, Pb, Al, and Cr.

Table 4 shows the leaching test for lightweight aggregates LWA1 and LWA2. Based on the presented results, it can be concluded that the toxic heavy metals such as Pb, Cd, Zn, Cr, Ni, and Cu were below the detection limits (DL) of the ICP-AES method.

Stabilization of heavy metals in lightweight aggregates (LWA1, LWA2) is due to the fact that the basic material of the aggregate is clay in which the main mineral components are beidellite, illite, and kaolinite. These are aluminosilicates of calcium, sodium, and magnesium, which due to their crystalline structure have a sorption ability of heavy metals. Ionic radius of heavy metals is so small that they can move freely in beidelite lattice, and exchangeable cations such as Na^+^ and Ca^2+^ can easily be exchanged for heavy metals. Also, calcium, sodium, potassium, magnesium, and iron ions present in illite and kaolinite occurring in the clay can be replaced by heavy metals. Thus, the crystal structure of these minerals allows to embedding of other elements, for example, heavy metal into them.

The stabilization process of heavy metals in lightweight aggregates is caused by a thermal synthesis reaction in the solid phase. This synthesis takes place at a temperature lower than the melting point of ingredients, and as a result of this reaction, new crystal structures are formed. Factors determining the reaction of the solid phase are among other diffusion, contact between the reactants, and the rate of nucleation of a new phase. The accumulated contact with each other grains are bonded after heating them to a suitable temperature. Then, the conversion of free-flowing powder in a solid polycrystal takes place. During sintering simultaneously occur phase changes, chemical reactions, and changes of microstructures. The sintering process to bonding of the heavy metal compounds in the aluminosilicate structure causes the heavy metal atoms to bind to other atoms at the molecular level.

The heavy metal crystallization means that ions such as Zn, Ni, Cu, Cr, and Ti could permeate into the aluminosilicate or silicate structure of the lightweight aggregates during the sintering process (Ho and Evans 2000). This heat-induced transformation to a crystalline state is advantageous and causes the long-term stability of these metals, because the crystalline solids have an improved capacity to bind heavy metals (Cheng and Chen 2003).

The reactions between sludge and clays constitute the dominant mechanism of sludge encapsulation (forming vitreous phase) (Magalhaes et al. 2004).

**Fig. 6:**
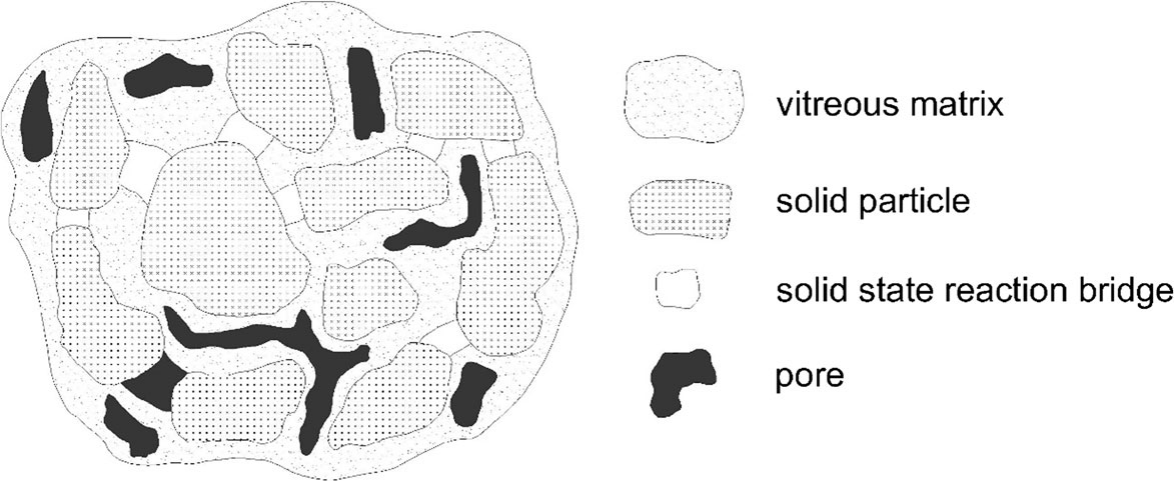
The microstructure scheme of ceramic materials that contain sewage sludges in their initial materials (based on Cusidó and Cremades 2012). The porosity (in *black*) caused by the thermal destruction of organic matter. The vitreous phase binds mostly heavy metals contained in both sludges and clays (mechanisms of macroencapsulation)

Inerting capacity of sintered aggregates results from a partial glass transition of minerals in the ceramic matrix, which contains most of the heavy metals. Organic matter in sintering process is totally destroyed that causes the high porosity of the microstructure (Fig. 6).

According to Cusidó and Cremades (2012). the presence of various sludges in ceramic materials increases neutralization inerting of heavy metals in comparison to a ceramic product made of 100 % of clay. Perhaps the combustion of organic matter thoroughly mixed clays causes a local increase of the temperature, which may promote the inertization of heavy metals in the crystal lattice.

Sintering of samples at temperature above the 1150 °C promotes the formation of a liquid phase and then crystalline phases. This leads to an increase of efficiency of solidification of heavy metals which are bonded in the crystal structures (Xu et al. 2010). This indicates that it is possible to use the tested aggregate as a completely ecological building material of significant value. Heavy metal compounds present in sewage sludge are built into the structure of lightweight aggregates.

## Discussion and conclusion

Sewage sludge from the municipal sewage treatment plant contains a considerable amount of heavy metals and is an efficient waste for the production of lightweight aggregate. Phase and microstructural studies as well as the leaching of heavy metals from aggregates have been conducted to prove this. The elements’ content in water extracts showed that the presence of metals is much smaller than is acceptable.

Important in presented leaching ability studies is that compounds of heavy metals present in sewage sludge are built into the structure of the resulting aluminosilicate where they are stable. As a result of the experiments, compounds are formed which occur in the natural environment (quartz, hematite) and as a product of calcination of aluminosilicates (mullite). Therefore, there is no risk of leaching and migration of those elements into the environment.

Lightweight aggregates derived from sewage sludge (LWA1 and LWA2) are valuable and safe building materials. Different firing temperatures (1100 and 1150 °C) allow one to obtain aggregates with different physical and mechanical properties. Reducing the temperatures by 50 °C has an effect on the economic aspect of creating LWA1 aggregates. With higher firing temperatures of LWA2 (1150 °C), lower specific, apparent, and bulk densities were obtained for these materials compared to aggregates fired at a lower temperature (LWA1). This is due to a higher pore volume which arises as a result of degassing of the organic matter present in the sewage sludge. This organic matter acts as a swelling agent. However, the densities of aggregates LWA1 and LWA2 meet standards’ requirements. LWA2 aggregates have a greater porosity (*P* = 66 %) and water absorption (*WA*_*24*_ = 16.2 %) and a much lower crushing strength (*S* = 0.79 MPa) than the aggregates of LWA1 (*P* = 60 %, *WA*_*24*_ = 14.4 %, *S* = 4.64 MPa). This is due to the formation of a more glassy coating on the surface and the inside of LWA2 aggregates and higher pore spaces. Between these aggregates, a clear difference in the size of pores can be observed. Additionally, between both tested aggregates, a clear difference in the size of pores was recorded. LWA2 aggregates have a larger pore size (5–90 μm) than LWA1 (5–30 μm) and the commercial aggregate of LWA0 (5–18 μm). Furthermore, LWA2 has similar physical and mechanical parameters as commercial aggregates available in Poland (LIAPOR or LECA WEBER). The absorptivity of LWA1 and LWA2 aggregates is in the range of 14.4–16.2 % and is comparable to the absorptivity of the Lytag aggregate (17 %) (Weinecke and Faulkner 2002) and the commercial aggregate of LIAPOR (10–20 %). Absorptivity values of other commercial aggregates are higher (30–40 % for LECA WEBER and 20–30 % for LWA0).

The values obtained for the compressive strength of tested aggregates (0.79 and 4.64 MPa) are satisfactory and are within range of the international standard values for solid waste materials used for ground leveling (>0.44 MPa) (Stegeman and Cot’e 1996). LWA1 aggregate (similar as LECA WEBER), despite the lower strength (0.79 MPa), and LWA2 can be used for insulation of floors, ceilings, roofs, vault fillings, drainages, concrete not carrying loads of insulation and insulation of pipelines and tanks in the ground.

The tested samples of lightweight aggregates (LWA1, LWA2) have a wide temperature interval, which is significantly higher than 50 °C. This is a positive result because melted granules are not sticking and the same as melted clay does not flood the oven.

Lightweight aggregates can be used in the production of elements filling the wooden frame or as blocks for building curtain wall.
